# Synthesis of Uracil-Iodonium(III) Salts for Practical Utilization as Nucleobase Synthetic Modules

**DOI:** 10.3390/molecules24173034

**Published:** 2019-08-21

**Authors:** Naoko Takenaga, Takumi Hayashi, Shohei Ueda, Hiroyuki Satake, Yoichi Yamada, Tetsuya Kodama, Toshifumi Dohi

**Affiliations:** 1 Faculty of Pharmacy, Meijo University, 150 Yagotoyama, Tempaku-ku, Nagoya 468-8503, Japan; 2 College of Pharmaceutical Sciences, Ritsumeikan University, 1-1-1 Nojihigashi, Kusatsu, Shiga 525–8577, Japan; 3 Department of Applied Chemistry, College of Life Sciences, Ritsumeikan University, 1-1-1 Nojihigashi, Kusatsu, Shiga 525–8577, Japan; 4 Department of Liberal Arts and Sciences, Kanagawa University of Human Services, 1-10-1 Heisei-cho, Yokosuka, Kanagawa 238-8522, Japan; 5 School of Pharmacy, Shujitsu University, 1-6-1 Nishigawara, Naka-ku, Okayama 703-8516, Japan; 6 Graduate School of Pharmaceutical Sciences, Nagoya University, Furo-cho, Chikusa-ku, Nagoya 464-8601, Japan

**Keywords:** hypervalent compound, iodonium salt, nucleobase, uracil

## Abstract

Iodonium(III) salts bearing uracil moieties have recently appeared in the literature, but their structural scope and utilization are limited because of their hygroscopic characteristics. In this study, we describe our detailed investigations for synthesizing a series of uracil iodonium(III) salts derived with various structural motifs and counterions. These new compounds have been utilized as attractive synthetic modules in constructing functionalized nucleobase and nucleosides.

## 1. Introduction

**Figure 1:**
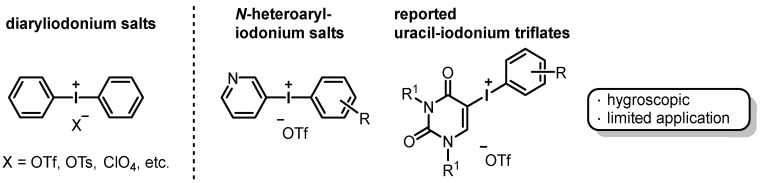
Conventional diaryliodonium(III) salts and their new heterocyclic series.

Diaryliodonium(III) salts, one of the useful and important classes of hypervalent iodine compounds, have a wide range of applications, such as active bactericides, benzyne precursors, and arylation reagents for reacting a wide range of nucleophiles—even under metal-free conditions [1,2,3,4,5,6,7,8]. *N*-Heteroaryliodonium salts (Figure 1, center), as well as conventional diaryliodonium salts (Figure 1, left), have also received considerable attention on account of their importance as versatile arylation reagents [9,10]. Classically, *N*-heteroaryliodonium salts could be obtained via the treatment of unstable vinyliodonium(III) dichloride with aryl lithium reagents [11,12,13]. The stepwise synthesis of 3-pyridyl(aryl)iodonium(III) salts via the corresponding 3-pyridyl-iododichloride has also been reported [14]. Recently, Olofsson et al. reported the one-pot synthesis of *N*-heteroaryliodonium triflates from the corresponding *N*-heteroaryliodides by the reaction toward arenes [15]. Although this approach is a versatile and reliable synthesis method of *N*-heteroaryliodonium triflates, there were still some limitations in the scope of substrates.

Nucleobases are important substructures in biologically active compounds [16,17], and the introduction of such moiety into iodonium(III) salts would be of high utility in organic synthesis. In 1998, Kim et al. reported the uracil-5-ylphenyliodonium(III) triflates (Figure 1, right) prepared by the reaction of 5-tributylstannylated uracils with aryl(cyano)iodonoium triflate. They also demonstrated the preparation of uracil-5-ylphenyliodonium(III) triflates via the reaction of unfunctionalized uracil with phenyliodine(III) diacetate (PIDA) in the presence of triflic acid, and applied it to the palladium-catalyzed alkenylations [18,19]. Recently, the Gaunt research team prepared heteroaryl-uracil-iodonium(III) triflates using *m*CPBA (*m*-chloroperbenzoic acid) and triflic acid as an oxidant and counterion, respectively, and then utilized these synthesized salts for organocatalytic arylation of aldehydes [20]. However, the isolation and application of uracil-iodonium(III) salts remained mostly limited to treating triflate (^−^OTf) salts [18,19,20,21,22], and the relationship between their stability and the structural feature of uracil-iodonium(III) salts has still not been sufficiently explored. This is likely because of their hygroscopic characteristics causing gradual decomposition, as claimed in previous studies [20]. In this context, we recently developed a series of stable uracil-iodonium(III) salts with various structural motifs and counterions that are suitable for isolation and storage [23]. Herein, we report the details of the preparation of further variation of uracil-iodonium(III) salts, together with their new applications.

## 2. Results and Discussions

**Scheme 1:**
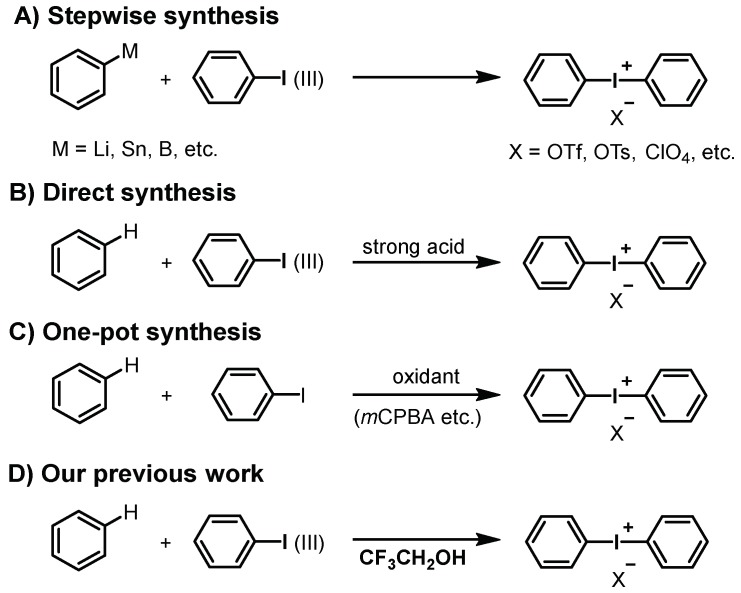
Reported synthetic routes of diaryliodonium(III) salts. *m*CPBA, *m*-chloroperbenzoic acid.

Synthetic methods and broad applications of conventional diaryliodonium(III) salts have been intensively investigated [5,6,7,8]. Synthetic routes to diaryliodonium(III) salts typically involve stepwise methods using a variety of organometallic nucleophiles, such as lithio-, silyl-, stannyl-, and boryl-arenes, instead of simply arenes themselves (Scheme 1A) [24,25,26,27]. To shorten the synthetic route, direct methods via electrophilic substitution were partially achieved for a limited number of aromatic compounds and counterions using the iodine(III) compounds activated by strong acids, or [hydroxy(tosyloxy)iodo]arenes; however, it led to only moderate yields and low regioselectivities (Scheme 1B) [28]. More recently, the synthesis of diaryliodonium(III) triflates via a straightforward approach, starting from iodoarenes or aromatic compounds, has been explored with the use of stoichiometric cooxidants such as *m*CPBA (Scheme 1C) [29]. As an environmentally-benign transformation utilizing the unique character of fluoroalcohol media, we established the direct, waste-free method for preparation of diaryliodonium(III) salts from a variety of arenes using hypervalent iodine(III) reagents (Scheme 1D) [30].

In a pilot experiment, using 1,3-dimethyluracil **1a** as a substrate, we examined the synthesis of uracil-aryliodonium triflates bearing electron-donating or electron-withdrawing aryl moieties in order to determine the influence of the substituent on the benzene ring (Table 1). PIDA derivatives bearing different para-substituents **2a**–**e** were first used for a protocol previously reported by Kim et al. [18]. The reported triflate **3aa-OTf** was produced in a seemingly good yield, and afterwards, the reaction was precipitated as fine white powder in diethyl ether. However, we became aware that this triflate **3aa-OTf** was severely hygroscopic and could not be easily isolated and stored during the experiment without strict care against air and moisture (Entry 1). Similar chemical behavior was previously noted by Gaunt et al., who reported that some uracil-aryliodonium(III) triflates are very difficult to handle and should be abruptly dried under a vacuum [20]. Moreover, triflate **3ab-OTf**, which was generated from *p*-tolyl-PIDA **2b**, was unstable, and its preparation thus resulted in decomposition during the reaction and workup. In contrast, the triflates **3ac-OTf**, **3ad-OTf**, and **3ae-OTf**, having an electron-withdrawing aryl moiety, were isolated in moderate to high yields as fine powders (Entries 3–5). Decreasing the amount of TfOH from two to one equivalent lowered the product yield (Entry 6). Considering the result from this screening, it seems that the presence of an electron-donating group decreased the stability of the uracil-aryliodonium triflates. In fact, uracil-iodonium(III) salts applied in different preparative methods were usually limited to the triflates with electron-deficient aryl moieties [20,21], which is in good accordance with our present observations.

**Scheme 2:**
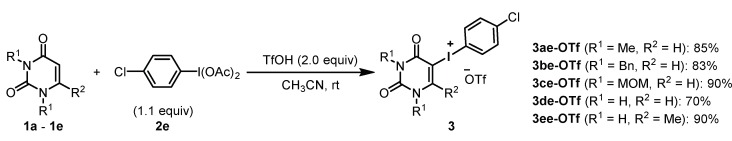
Availability of uracil *N*-protecting groups of uracil-iodonium(III) triflates.

We then confirmed the suitability of *N*-protecting groups for the uracil molecule in the reaction with 4-ClC_6_H_4_I(OAc)_2_ (**2e**) to give the corresponding iodonium(III) triflates (Scheme 2). Using acetonitrile as a solvent instead of dichloromethane allowed the triflate **3ae-OTf** to be obtained with a slightly higher yield of 85%. The *N*-protected uracils containing benzyl (R^1^ = Bn, R^2^ = H; **1b**) and methoxymethyl (R^1^ = MOM, R^2^ = H; **1c**) groups were treated for the reaction conditions, producing the corresponding iodonium(III) triflates **3be-OTf** and **3ce-OTf** in high yields. The conditions could also be applied to uracil itself (R^1^ = H, R^2^ = H; **1d**) and 6-methyluracil (R^1^ = H, R^2^ = Me; **1e**), generating 70% and 90% yields in the triflates **3de-OTf** and **3ee-OTf**, respectively. In the case of thymine, or 5-methyluracil as a substrate, the precipitate was not detected.

In general, the chemical and physical properties of iodonium(III) salts strongly depend on the nature of both the aryl moiety and the anionic counterpart. We then began to prepare uracil-iodonium(III) derivatives carrying different types of counterions, as shown in Table 2. Among the reported various protocols for iodonium(III) salt synthesis, we conducted the dehydrative condensation of uracil with a Koser-type reagent (ArI(OH)OTs) for the preparation of uracil-aryliodonium(III) tosylate in fluoroalcohol medium, according to our established procedure (Scheme 1D) [30]. The reaction was thus performed with 1,3-dimethyluracil **1a** and stoichiometric 4-ClC_6_H_4_I(OH)OTs **2f** in 2,2,2-trifluoroethanol (TFE), which successfully gave the desired tosylate **3af-OTs** in a high yield (Entry 1). The use of 1,1,1,3,3,3-hexafluoroisopropanol (HFIP) instead of TFE resulted in a small decrease in product yield (Entry 2). An extensive number of Koser-type reagents are readily available, and their variations in the dehydrative condensation can expand the structures of the obtained iodonium(III) products. One example of this extension is the preparation of the mesylate salt, **3ag-OMs** (Entry 3). Similar modification of the counterion was possible by the utilization of the PIDA derivative **2e**, which generated (+)-10-camphorsulfonate **3ae-OCs** (Entry 4). Trifluoroacetate and perchlorate anions were conveniently introduced as counterions to the products, **3ae-OCOCF_3_** and **3ae-ClO_4_**, under similar reaction conditions (Entries 5 and 6). We also found that among various counterions, the uracil-iodonium(III) tosylates demonstrated especially high stability for ease of handling; thus, we sought to prepare uracil-iodonium(III) tosylates carrying different types of aryl moieties (Entries 7–10). The tosylate salt **3ah-OTs** was non-hygroscopic, stable under air, and tolerable to prolonged storage, while the corresponding triflate **3aa-OTf** was hygroscopic and difficult to handle (Table 2, Entry 7 versus Table 1, Entry 1). Other tosylates having 4-nitrophenyl and 4-trifluoromethylphenyl moieties could be synthesized in the same manner (Entries 8 and 9). This protocol in fluoroalcohol was also effective for the preparation of triflates (Entries 10–12).

**Scheme 3:**
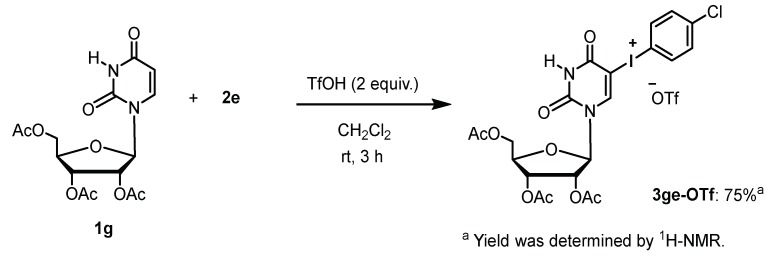
Preparation of uracil-iodonium(III) salts **3ge-OTf**.

Aiming at developing useful synthetic modules in nucleoside chemistry, we then attempted to synthesize iodonium(III) salts bearing 2,3,5-tri-*O*-acetyluridine (Scheme 3). Similarly, to a stirred solution of 2,3,5-tri-*O*-acetyluridine **1g** and 4-chloroiodobenzene diacetate **2e** in dichloromethane, a solution of TfOH in dichloromethane was added dropwise at room temperature. After completion of the reaction, the solvent was removed and then precipitated by adding Et_2_O through stirring of the desired salt **3ge-OTf**. The salt **3ge-OTf** was stable as fine powder for at least 1 h with the care of moisture. However, as time passed, the hygroscopic salt **3ge-OTf** melted and became gummy-like material. To overcome the instability of this salt **3ge-OTf**, we further examined the counterion exchange [31] and other solvents; unfortunately, all these attempts did not lead to the improvement of the stability of the salt **3ge-OTf**.

**Scheme 4:**
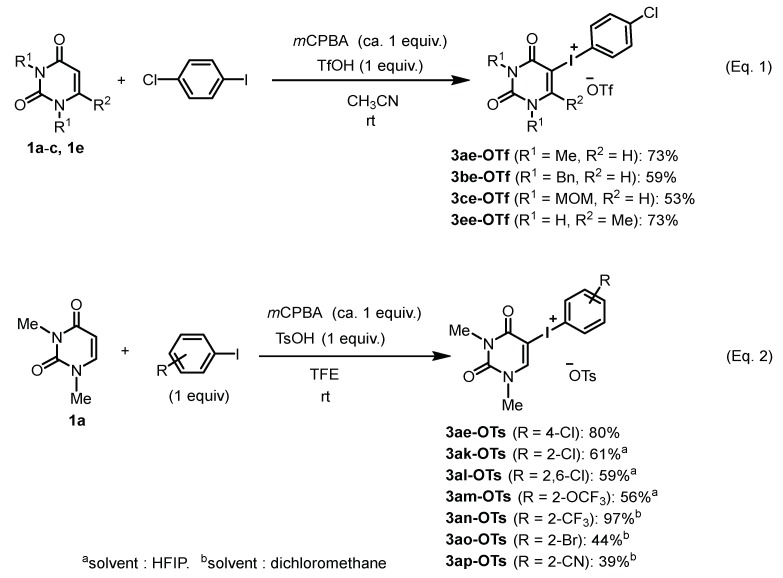
Preparation of uracil-iodonium(III) salts using *m*CPBA.

We next performed the one-pot synthesis of uracil-iodonium(III) salts from aryl iodides and uracils utilizing *m*CPBA as an oxidant (Scheme 4). The triflates **3ae-OTf**—**3ce-OTf** and **3ee-OTf** mentioned above could be prepared in moderate to high yields using an equimolar amount of 4-chloroiodobenzene (Equation (1)). Likewise, a variety of tosylate salts bearing a broad range of aromatic moieties, such as 4-chlorobenzene, 2-chlorobenzene, 2,6-dichlorobenzene, 2-trifluoromethoxybenzene, 2-trifluoromethylbenzene, 2-bromobenzene, and 2-cyanobenzene, can be readily prepared (Equation (2)). In some cases, using HFIP or CH_2_Cl_2_ instead of TFE could produce tosylate salts in higher yields.

**Scheme 5:**
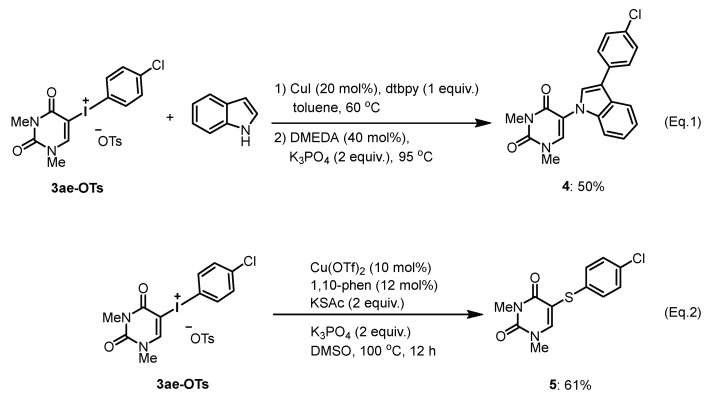
Copper-catalyzed diarylation and C–S coupling.

Application studies of the obtained uracil-iodonium(III) salts **3** were then undertaken to exemplify their utility in several reactions. The selected examples are shown in Scheme 5. Thus, the Cu(I)-catalyzed sequential C−H and N−H arylation of indole [21] by iodonium triflate **3ae-OTs** produced the indoyl uracil **4** in moderate yield (Scheme 5, Equation (1)). On the other hand, Cu(II)-catalyzed sulfide construction through a sulfur-iodine exchange protocol [32] of iodonium(III) triflate **3ae-OTs** with potassium thioacetate generated **5** in 61% yield (Equation (2)).

Furthermore, as shown in Table 3, the reactive heteroaryne analogues, generated from the uracil iodonium(III) salts **3** by deprotonation using lithium bis(trimethylsilyl)amide (LiHMDS) [33], could be trapped with furan **6a**—producing the [4 + 2] cycloaddition products **7a**. When uracil-iodonium(III) tosylate **3ae-OTs**, including a 4-chlorophenyl group, was applied to the reaction, the cyclization product **7a** was obtained in 31% yield (Entry 1). Although the use of 4-trifluoromethylphenyliodonium(III) tosylate **3aj-OTs** led to an inferior product yield (Entry 2), the corresponding tosylate **3ak-OTs**—including a 2-chlorophenyl group—improved the yield result of this reaction (Entry 3). We then attempted several 2-substituted aryl iodonium(III) tosylates, such as 2,6-dichlorophenyl **3al-OTs** (Entry 4), 2-fluorophenyl **3aq-OTs** (Entry 5), 2-trifluoromethoxy **3am-OTs** (Entry 6), and 2-trifluoromethylphenyl **3an-OTs** (Entry 7). Among the various uracil iodonium(III) salts tested, **3an-OTs** was found to be most promising.

To confirm the scope of this reaction, we examined a variety of arynophiles **6** using the optimized experimental procedure (Table 4). The cycloaddition using tosylate **3an-OTs** with 2,5-dimethylfuran **6b** gave the expected product **7b** in a 42% yield (Entry 2). In the case of *N*-substituted pyrroles **6c**-**e**, the reactions also proceeded smoothly and finished within 3 h to furnish the corresponding products **7c**–**e** in acceptable yields (Entries 3–5). When diphenylisobenzofuran **6f** was subjected to the reaction at 40 °C, the cycloadduct **7f** was produced and isolated in 55% (Entry 6).

**Scheme 6:**
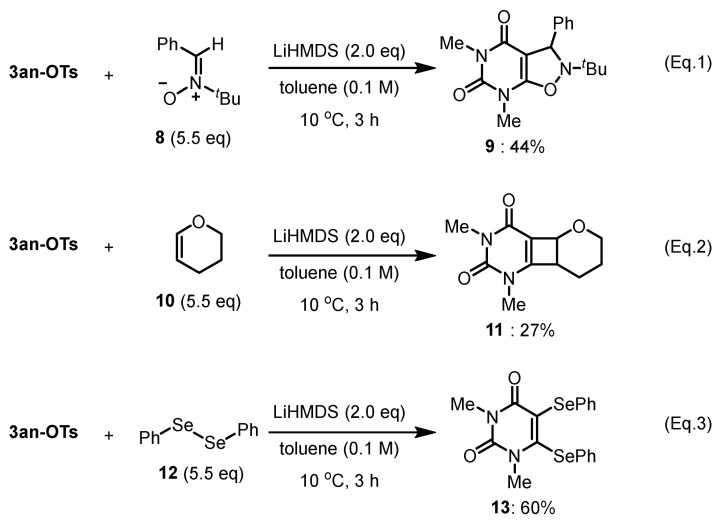
[3 + 2] and [2 + 2] cyclizations, and σ-bond insertion using uracil-iodonium(III) salt **3an-OTs**.

Having established the [4 + 2] cycloaddition conditions using the **3an-OTs** above, we were able to extend the reactivity of the uracil iodonium(III) salts to other types of cycloadditions. The representative results are given in Scheme 6. Thus, the reaction of **3an-OTs** with *N*-*tert*-Butyl-phenyl nitrone **8** [34] afforded a [3 + 2] annulated product **9** (Equation 1). The cycloaddition of 3,4-dihydro-2*H*-pyran **10** [35] as an alkynophile yielded the [2 + 2] annulated product **11** (Equation 2). When diphenyldiselenide **12** [36] was subjected to the reaction conditions, σ-bond insertion occurred and the 5,6-difunctionalized product **13** was obtained in a moderate yield (Equation (3)).

**Figure 2:**
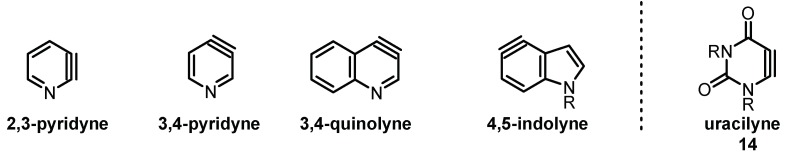
Reported heteroarynes and uracil-heteroaryne analog **14**.

On the basis of the experimental results in Table 3 and Table 4 and Scheme 6, we would speculate that the reaction mechanisms for these cycloadditions and σ-bond insertion involve the formation of the heteroaryne analogue of uracil, as in uracilyne **14** (Figure 2). LiHMDS can abstract the C*sp*^2^ uracil ring hydrogen of the iodonium(III) salts **3**. Owing to the exceptionally high leaving group ability of the aryl iodanyl group [37], the facile elimination of 2-substituted iodobenzene for generation of uracilyne **14** was possible. This reactive alkyne species **14**, having a highly strained and distorted C≡C bond [38,39,40,41], can react with the arynophiles **6** to give the corresponding cycloadducts **7**. At present, there is no report on the successful generation of cyclic uracil alkyne **14** (uracilyne, Figure 2) and its vicinal difunctionalization. The aryne chemistry has a synthetic advantage for the multi-functionalization of aromatic rings in a single operation [42]. Heteroarynes [43,44], such as pyridyne and indolyne (Figure 2), are thus regarded as attractive tools for the construction of multi-functionalized heteroarene derivatives [43,44,45,46,47,48,49]. When compared with the benzyne chemistry, the application of heteroarynes is still not fully investigated. For example, the treatment of halouracils with a variety of strong bases failed to generate **14** [43]. Similarly, uracilyne **14** could not be generated from iodouracil [50]. In a more recent report, Garg et al. attempted to generate pyrimidyne from silyltriflate precursors, but failed in the result [51].

## 3. Conclusions

In conclusion, we demonstrated detailed investigations and new application protocols concerning the synthesis of uracil-iodonium(III) salts and their use as attractive synthetic modules in constructing unique functionalized molecules containing nucleobases. We found that the stabilizing effect of the tosylate moiety facilitates the preparation of uracil-iodonium(III) salts carrying different types of aryl moieties. These insights can encourage the utilization of uracil iodonium(III) salt as a useful building block in organic synthesis. Further investigations on the utilization of uracil iodinium(III) salts are underway in our research groups.

## 4. Experimental Section

Melting points (mp) are uncorrected. The ^1^H-NMR (and ^13^C-NMR) spectra of the products were recorded by JEOL JMN-300 or Bruker Avance III 600 spectrometer operating at 400 or 600 MHz (100 or 150 MHz for ^13^C-NMR) in CD_3_OD or CDCl_3_ at 25 °C with tetramethylsilane as the internal standard. The data are reported as follows: chemical shift in part par million (δ), multiplicity (s = singlet, d = doublet, t = triplet, q = quartet, br = broad singlet, m = multiplet), integration, and coupling constant (Hz). The infrared spectra (IR) were obtained using a Hitachi 270-50 spectrometer and the absorptions are reported in reciprocal centimeters (cm^−1^) for representative peaks. High resolution mass spectra were measured with a Thermo Scientific Exactive Plus Orbitrap. Phenyliodine(III) diacetate (PIDA; **2a**) and PhI(OH)OTs (HTIB, Koser’s reagent; **2h**) are commercially available compounds and were used as received. Other hypervalent iodone(III) reagents **2b**–**g** and **2i**–**j** were synthesized from the corresponding commercial iodoarenes by oxidations, according to the literature procedures [52,53,54,55]. The *N*-protected uracils **1b**, **1c**, and **1f** were prepared from uracil by the known methods [56,57,58]. Solvents and all other starting materials were obtained from commercial suppliers and used without further purification.

### 4.1. General Procedure for the Synthesis of Uracil-Iodonium(III) Triflates 

To a stirred solution of 1,3-dimethyluracil **1a** (0.50 mmol) and 4-chloroiodobenzene diacetate **2e** (0.55 mmol) in dichloromethane or acetonitrile (2 mL), a solution of trifluoromethanesulfonic acid (ca. 150 mg, 1.0 mmol, 2 equiv) in acetonitrile (1 mL) was added dropwise at room temperature, and the resulting slightly colored solution was stirred for 3 h. After addition of methanol (~2 mL), the solvents were removed under reduced pressure. The residue was then treated with diethyl ether with stirring for precipitation of the iodonium(III) salt. The precipitate was filtered and dried in vacuo to give a pure iodonium(III) salt in powder form.

*(1,3-Dimethyl-2,4-dioxo-1,2,3,4-tetrahydropyrimidin-5-yl)(4-nitrophenyl)iodonium triflate* (**3ac-OTf**). A white powder, m.p. 212–213 °C. IR (KBr) cm^−1^: 1716, 1663, 1352, 1248, 1023, 632. ^1^H-NMR (600 MHz, CD_3_OD) δ 3.34 (s, 3H), 3.50 (s, 3H), 8.33 (d, 2H, *J* = 9.0 Hz), 8.39 (d, 2H, *J* = 9.0 Hz), 8.97 (s, 1H) ppm. ^13^C-NMR (150 MHz, CD_3_OD) δ 28.4, 37.0, 88.2, 120.3, 125.9, 136.2, 150.2, 150.9, 155.0, 159.4 ppm. HRMS (FAB): Calcd. for C_12_H_11_IN_3_O_4_ [M − OTf]^+^: 387.9789, found: 387.9791.

*(1,3-Dimethyl-2,4-dioxo-1,2,3,4-tetrahydropyrimidin-5-yl)(4-(trifluoromethyl)phenyl)iodonium triflate* (**3ad-OTf**). A white powder, m.p. 190–191 °C. IR (KBr) cm^−1^: 1723, 1680, 1614, 1325, 1229, 1025, 639. ^1^H-NMR (600 MHz, CD_3_OD) δ 3.34 (s, 3H), 3.49 (s, 3H), 7.85 (d, 2H, *J* = 8.4 Hz), 8.35 (d, 2H, *J* = 8.4 Hz), 8.96 (s, 1H) ppm. ^13^C-NMR (150 MHz, CD_3_OD) δ 28.4, 37.0, 88.1, 118.6, 128.0, 128.1, 128.2, 135.7, 150.9, 154.8, 159.4 ppm. HRMS (FAB): Calcd. for C_13_H_11_F_3_IN_2_O_2_ [M − OTf]^+^: 410.9812, found: 410.9816.

*(4-Chlorophenyl)(1,3-dimethyl-2,4-dioxo-1,2,3,4-tetrahydropyrimidin-5-yl)iodonium triflate* (**3ae-OTf**). A white powder, m.p. 154–155 °C. IR (KBr) cm^−1^: 1717, 1662, 1514, 1251, 1227, 1024, 631. ^1^H-NMR (600 MHz, CD_3_OD) δ 3.34 (s, 3H), 3.48 (s, 3H), 7.55 (d, 2H, *J* = 9.0 Hz), 8.13 (d, 2H, *J* = 9.0 Hz), 8.92 (s, 1H) ppm. ^13^C-NMR (150 MHz, CD_3_OD) δ 29.8, 38.3, 89.5, 113.6, 133.0, 138.1, 140.4, 152.3, 155.9, 160.8 ppm. HRMS (FAB): Calcd. for C_12_H_11_ClIN_2_O_2_ [M − OTf]^+^: 376.9548, found: 376.9546.

*(2,4-Dioxo-1,2,3,4-tetrahydropyrimidin-5-yl)(4-chlorophenyl)iodonium triflate* (**3de-OTf**). A white powder, m.p. 191-192 °C. IR (KBr) cm^−1^: 1738, 1670, 1366, 1216, 1164. ^1^H-NMR (600 MHz, CD_3_OD) δ 7.56 (d, 2H, *J* = 9.0 Hz), 8.10 (d, 2H, *J* = 9.0 Hz), 8.70 (s, 1H) ppm. ^13^C-NMR (150 MHz, CD_3_OD) δ 90.9, 113.5, 133.0, 137.9, 140.4, 143.5, 152.2, 154.6, 161.6 ppm. HRMS (DART): Calcd. for C_10_H_7_ClIN_2_O_2_ [M − OTf]^+^: 348.9235, found: 348.9236.

### 4.2. General Procedure for the Synthesis of Uracil-Iodonium(III) Tosylates

To a solution of 1,3-dimethyluracil **1a** (0.20 mmol) in 2,2,2-trifluoroethanol (TFE) (3 mL), (4-chlorophenyl)(hydroxy)iodonium tosylate **2f** (0.22 mmol) was added and it was stirred at room temperature. After completion of the reaction, the solvent was removed under vacuum. The product was then precipitated by the addition of Et_2_O with stirring. The precipitate was filtered to give uracil-iodonium(III) salt **3af-OTs** as a white powder.

*(4-Chlorophenyl)(1,3-dimethyl-2,4-dioxo-1,2,3,4-tetrahydropyrimidin-5-yl)iodonium tosylate* (**3af-OTs**). A white powder, m.p. 186–187 °C. IR (KBr) cm^−1^: 1715, 1656, 1615, 1214, 1169, 997, 682. ^1^H-NMR (600 MHz, CD_3_OD) δ 2.37 (s, 3H), 3.33 (s, 3H), 3.47 (s, 3H), 7.23 (d, 2H, *J* = 7.8 Hz), 7.54 (d, 2H, *J* = 9.0 Hz), 7.69 (d, 2H, *J* = 7.8 Hz), 8.12 (d, 2H, *J* = 9.0 Hz), 8.92 (s, 1H) ppm. ^13^C-NMR (150 MHz, MeOD) δ 19.9, 28.4, 36.9, 88.2, 112.3, 125.5, 128.4, 131.6, 136.7, 139.0, 140.3, 142.2, 150.9, 154.5, 159.4 ppm. HRMS (FAB): Calcd. for C_12_H_11_ClIN_2_O_2_ [M − OTs]^+^: 376.9548, found: 376.9569.

*(4-Chlorophenyl)(1,3-dimethyl-2,4-dioxo-1,2,3,4-tetrahydropyrimidin-5-yl)iodonium mesylate* (**3ag-OMs**). A white powder, m.p. 185–186 °C. IR (KBr) cm^−1^: 1739, 1715, 1662, 1218, 1158, 1033. ^1^H-NMR (600 MHz, CD_3_OD) δ 2.70 (s, 3H), 3.34 (s, 3H), 3.49 (s, 3H), 7.56 (d, 2H, *J* = 9.0 Hz), 8.13 (d, 2H, *J* = 9.0 Hz), 8.94 (s, 1H) ppm; ^13^C-NMR (150 MHz, CD_3_OD) δ 29.8, 38.3, 40.0, 90.0, 113.7, 133.0, 138.1, 140.4, 152.4, 156.0, 160.8 ppm. HRMS (FAB): Calcd. for C_12_H_11_ClIN_2_O_2_ [M − OMs]^+^: 376.9548, found: 376.9545.

*(4-Chlorophenyl)(1,3-dimethyl-2,4-dioxo-1,2,3,4-tetrahydropyrimidin-5-yl)iodonium (+)-10-camphorsulfonate* (**3ae-OCs**). A white powder, m.p. 194–195 °C. IR (KBr) cm^−1^: 1747, 1717, 1653, 1353, 1217, 1204. ^1^H-NMR (600 MHz, CD_3_OD) δ 0.87 (s, 3H), 1.15 (s, 3H), 1.41–1.44 (m, 1H), 1.61–1.63 (m, 1H), 1.91 (d, 1H, *J* = 14.4 Hz), 2.05–2.08 (m, 2H), 2.34–2.37 (m, 1H), 2.67–2.69 (m, 1H), 2.77 (d, 1H, *J* = 14.4 Hz), 3.31–3.33 (m, 1H), 3.36 (s, 3H), 3.51 (s, 3H), 7.57 (d, 2H, *J* = 9.0 Hz), 8.15 (d, 2H, *J* = 9.0 Hz), 8.96 (s, 1H) ppm. ^13^C-NMR (150 MHz, CD_3_OD) δ 20.1, 20.4, 25.8, 27.8, 29.8, 38.4, 43.6, 44.0, 59.6, 89.6, 113.7, 132.9, 138.1, 140.3, 152.4, 156.1, 160.9, 218.2 ppm. HRMS (FAB): Calcd. for C_12_H_11_ClIN_2_O_2_ [M − OCs]^+^: 376.9548, found: 376.9552.

*(4-Chlorophenyl)(1,3-dimethyl-2,4-dioxo-1,2,3,4-tetrahydropyrimidin-5-yl)iodonium trifluoroacetate* (**3ae-OCOCF_3_**). A white powder, m.p. 174–175 °C. IR (KBr) cm^−1^: 1714, 1652, 1609, 1191, 1137. ^1^H-NMR (600 MHz, CD_3_OD) δ 3.36 (s, 3H), 3.50 (s, 3H), 7.58 (d, 2H, *J* = 8.4 Hz), 8.14 (d, 2H, *J* = 8.4 Hz), 8.94 (s, 1H) ppm; ^13^C-NMR (150 MHz, CD_3_OD) δ 28.4, 36.9, 88.2, 112.3, 130.2, 131.6, 136.6, 138.8, 139.0, 150.9, 154.4, 159.4 ppm. HRMS (FAB): Calcd. for C_12_H_11_ClIN_2_O_2_ [M − OCOCF_3_]^+^: 376.9548, found: 376.9556.

*(4-Chlorophenyl)(1,3-dimethyl-2,4-dioxo-1,2,3,4-tetrahydropyrimidin-5-yl)iodonium perchlorate* (**3ae-ClO_4_**). A white powder, m.p. 134–135 °C. IR (KBr) cm^−1^: 1720, 1653, 1610, 1120, 1083. ^1^H-NMR (600 MHz, CD_3_OD) δ 3.34 (s, 3H), 3.49 (s, 3H), 7.54 (d, 2H, *J* = 8.4 Hz), 8.13 (d, 2H, *J* = 8.4 Hz), 8.91 (s, 1H) ppm; ^13^C-NMR (150 MHz, CD_3_OD) δ 29.8, 38.4, 89.4, 113.6, 133.0, 138.1, 140.4, 152.4, 156.0, 160.9 ppm; HRMS (FAB): Calcd. for C_12_H_11_ClIN_2_O_2_ [M − ClO_4_]^+^: 376.9548, found: 376.9563.

### 4.3. Reaction of Uracil-Iodonium(III) Tosylates with Arynophiles

In a flame-dried flask, under nitrogen, to a mixture of iodonium salt **3an-OTs** (0.50 mmol) and furan **6a** (2.75 mmol, 5.5 equiv) in toluene (5 mL, 0.1 M) in an ice-cooled bath maintained at 10 °C, LiHMDS (0.77 mL (1.3 M in toluene), 1.0 mmol, 2.0 equiv) was dropwise added by syringe, and the mixture was stirred for 3 h. After completion of the reaction checked by TLC, the reaction mixture was quenched with an aqueous solution of ammonium chloride. The resultant biphasic solution was extracted with CH_2_Cl_2_, dried with solid sodium sulfate, and then concentrated. The residue was purified by column chromatography on silica gel using hexane-EtOAc as eluent to give **7a** as a white solid (40%).

*1,3-Dimethyl-5,8-dihydro-5,8-epoxyquinazoline-2,4(1H,3H)-dione* (**7a**). A white solid, mp 140–141 °C. IR: 2967, 2928, 1662, 1466, 1389, 1361, 1151 cm^−1^. ^1^H-NMR (400 MHz, CDCl_3_) δ 7.34 (dd, *J* = 5.6, 1.6 Hz, 1H), 7.01 (dd, *J* = 5.2, 2.0 Hz, 1H), 5.83–5.92 (m, 1H), 5.58–5.66 (m, 1H), 3.46 (s, 3H), 3.27 (s, 3H) ppm; ^13^C-NMR (100 MHz, CDCl_3_) δ 170.5, 158.2, 151.5, 148.0, 138.8, 118.6, 81.4, 81.0, 33.4, 28.2 ppm; HRMS (FAB): Calcd. for C_10_H_11_N_2_O_3_ [M + H]^+^: 207.0770, found: 207.0771.

The reactions of other substrates **3**, **8**, **10**, and **12** shown in Table 3 and Scheme 6 were performed by the same experimental procedures.

## Figures and Tables

**Table 1:**
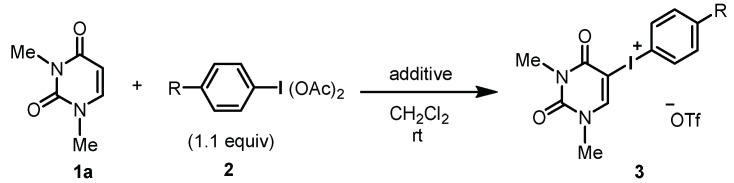
Influence of substituents on benzene ring of uracil-iodonium(III) triflates.

Entry	R	I (III)	TfOH	Product	Yield (%)
1	H	I(OAc)_2_ (**2a**)	2.0 equiv.	**3aa-OTf**	hygroscopic
2	Me	I(OAc)_2_ (**2b**)	2.0 equiv.	**3ab-OTf**	decomp.
3	NO_2_	I(OAc)_2_ (**2c**)	2.0 equiv.	**3ac-OTf**	56
4	CF_3_	I(OAc)_2_ (**2d**)	2.0 equiv.	**3ad-OTf**	75
5	Cl	I(OAc)_2_ (**2e**)	2.0 equiv.	**3ae-OTf**	78
6	Cl	I(OAc)_2_ (**2e**)	1.0 equiv.	**3ae-OTf**	19

**Table 2:**
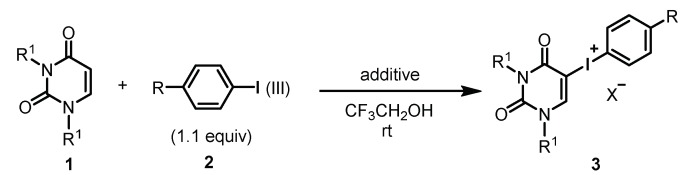
Facile synthesis of uracil-iodonium(III) salts with various counterions.

Entry	R^1^	R	I (III)	Additive	X	Product	Yield (%)
1	Me	4-Cl	I(OH)OTs (**2f**)	none	OTs	**3af-OTs**	98
2 ^a^	Me	4-Cl	I(OH)OTs (**2f**)	none	OTs	**3af-OTs**	89
3	Me	4-Cl	I(OH)OMs (**2g**)	none	OMs ^b^	**3ag-OMs**	74
4	Me	4-Cl	I(OAc)_2_ (**2e**)	(+)-10-CSA ^c^	(+)-10-OCs ^d^	**3ae-OCs**	74
5	Me	4-Cl	I(OAc)_2_ (**2e**)	CF_3_CO_2_H	OCOCF_3_	**3ae-OCOCF_3_**	72
6	Me	4-Cl	I(OAc)_2_ (**2e**)	HClO_4_	ClO_4_	**3ae-ClO_4_**	68
7	Me	H	I(OH)OTs (**2h**)	none	OTs	**3ah-OTs**	78
8	Me	4-NO_2_	I(OH)OTs (**2i**)	none	OTs	**3ai-OTs**	20
9	Me	4-CF_3_	I(OH)OTs (**2j**)	none	OTs	**3aj-OTs**	16
10	Me	4-Cl	I(OAc)_2_ (**2e**)	TfOH	OTf	**3ae-OTf**	98
11	Bn	4-Cl	I(OAc)_2_ (**2e**)	TfOH	OTf	**3be-OTf**	75
12	MEM	4-Cl	I(OAc)_2_ (**2e**)	TfOH	OTf	**3fe-OTf**	55

^a^ 1,1,1,3,3,3-hexafluoroisopropanol (HFIP) was used instead of 2,2,2-trifluoroethanol (TFE). ^b^ OMs = methanesulfonyloxy, ^c^ CSA = camphorsulfonic acid, ^d^ OCs = camphorsulfonyloxy.

**Table 3:**
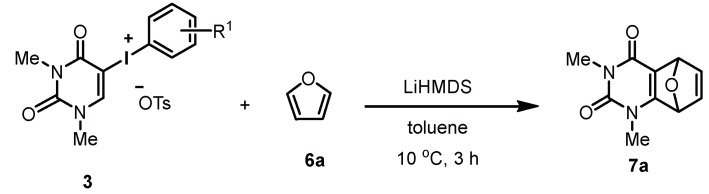
Optimization of furan addition to uracil ring with base activation of iodonium(III) salt **3** ^a^. LiHMDS, lithium bis(trimethylsilyl)amide.

Entry	R^1^	3	Yield (%) ^b^
1	4-Cl	**3ae-OTs**	31
2	4-CF_3_	**3aj-OTs**	20
3	2-Cl	**3ak-OTs**	37
4	2,6-Cl	**3al-OTs**	24
5	2-F	**3aq-OTs**	22
6	2-OCF_3_	**3am-OTs**	28
7	2-CF_3_	**3an-OTs**	40 ^c^

^a^ Reactions were performed using 2 equiv of LiHMDS and 5.5 equiv of furan **6a** at 10 °C in toluene (0.1 M). ^b^ Determined by ^1^H-NMR. ^c^ Isolated yield.

**Table 4:**
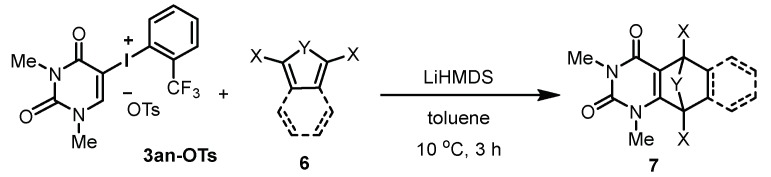
[4 + 2] Cycloaddition using uracil-iodonium(III) salt **3an-OTs** ^a^.

Entry	6	X	Y	Product 7	Yield (%)
	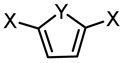				
1	**6a**	H	O	**7a**	40
2	**6b**	Me	O	**7b**	42
3	**6c**	H	NPh	**7c**	38
4	**6d**	H	NBoc	**7d**	45
5	**6e**	H	N-4-CF_3_C_6_H_4_	**7e**	42
6 ^b^	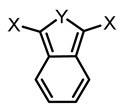 **6f**	Ph	O	**7f**	55

^a^ Reactions were performed using 2 equiv of LiHMDS and 5.5 equiv of arynophile **6** at 10 °C in toluene (0.1 M). ^b^ Reaction was performed at 40 °C.
